# Ensemble Deep Learning Model to Predict Lymphovascular Invasion in Gastric Cancer

**DOI:** 10.3390/cancers16020430

**Published:** 2024-01-19

**Authors:** Jonghyun Lee, Seunghyun Cha, Jiwon Kim, Jung Joo Kim, Namkug Kim, Seong Gyu Jae Gal, Ju Han Kim, Jeong Hoon Lee, Yoo-Duk Choi, Sae-Ryung Kang, Ga-Young Song, Deok-Hwan Yang, Jae-Hyuk Lee, Kyung-Hwa Lee, Sangjeong Ahn, Kyoung Min Moon, Myung-Giun Noh

**Affiliations:** 1 Department of Medical and Digital Engineering, Hanyang University College of Engineering, Seoul 04763, Republic of Korea; jonghyunlee1993@gmail.com; 2 Department of Pre-Medicine, Chonnam National University Medical School, 322 Seoyang-ro, Hwasun-eup, Hwasun-gun, Gwangju 58128, Republic of Korea; riobird@naver.com; 3 NetTargets, 495 Sinseong-dong, Yuseong, Daejeon 34109, Republic of Korea; 4 AMGINE, Inc., Jeongui-ro 8-gil 13, Seoul 05836, Republic of Korea; jkim325@aucklanduni.ac.nz; 5 Department of Convergence Medicine, Asan Medical Center, University of Ulsan College of Medicine, Seoul 25440, Republic of Korea; namkugkim@gmail.com (N.K.); tobeor3009@gmail.com (S.G.J.G.); 6 Division of Biomedical Informatics, Seoul National University Biomedical Informatics (SNUBI), Seoul National University College of Medicine, Seoul 03080, Republic of Korea; juhan@snu.ac.kr; 7 Department of Radiology, Stanford University School of Medicine, Stanford, CA 94305-5101, USA; sosal@stanford.edu; 8 Department of Pathology, Chonnam National University Medical School, Gwangju 61469, Republic of Korea; drydchoi@hanmail.net; 9 Department of Nuclear Medicine, Clinical Medicine Research Center, Chonnam National University Hospital, 671 Jebongno, Gwangju 61469, Republic of Korea; srkang@jnu.ac.kr; 10 Departments of Hematology-Oncology, Chonnam National University Hwasun Hospital, 322 Seoyangro, Hwasun 58128, Republic of Korea; drgyssong@gmail.com (G.-Y.S.); drydh1685@hotmail.com (D.-H.Y.); 11 Department of Pathology, Chonnam National University Hwasun Hospital and Medical School, 322 Seoyang-ro, Hwasun-eup, Hwasun-gun, Hwasun 58128, Republic of Koreamdkaylee@gmail.com (K.-H.L.); 12 Department of Pathology, Korea University Anam Hospital, Korea University College of Medicine, 73 Goryeodae-ro, Seongbuk-gu, Seoul 02841, Republic of Korea; vanitasahn@gmail.com; 13 Division of Pulmonary and Allergy Medicine, Department of Internal Medicine, Chung-Ang University Hospital, Chung-Ang University College of Medicine, Seoul 06973, Republic of Korea; 14 Artificial Intelligence, ZIOVISION Co., Ltd., Chuncheon 24341, Republic of Korea

**Keywords:** digital pathology, artificial intelligence, gastric cancer, lymphovascular invasion

## Abstract

**Simple Summary:**

Lymphovascular invasion (LVI) serves as a crucial predictor in gastric cancer, indicating an increased likelihood of lymph node spread and poorer patient outcomes. Detecting LVI(+) within gastric cancer histopathology presents challenges due to its elusive nature, leading to the proposal of a deep learning-based detection method using H&E-stained whole-slide images. Remarkably, both the classification and detection models demonstrated superior performance, and their ensemble exhibited outstanding predictive capabilities in identifying LVI areas. This innovative approach holds promise in precision medicine, potentially streamlining examinations and reducing discrepancies among pathologists.

**Abstract:**

Lymphovascular invasion (LVI) is one of the most important prognostic factors in gastric cancer as it indicates a higher likelihood of lymph node metastasis and poorer overall outcome for the patient. Despite its importance, the detection of LVI(+) in histopathology specimens of gastric cancer can be a challenging task for pathologists as invasion can be subtle and difficult to discern. Herein, we propose a deep learning-based LVI(+) detection method using H&E-stained whole-slide images. The ConViT model showed the best performance in terms of both AUROC and AURPC among the classification models (AUROC: 0.9796; AUPRC: 0.9648). The AUROC and AUPRC of YOLOX computed based on the augmented patch-level confidence score were slightly lower (AUROC: −0.0094; AUPRC: −0.0225) than those of the ConViT classification model. With weighted averaging of the patch-level confidence scores, the ensemble model exhibited the best AUROC, AUPRC, and F1 scores of 0.9880, 0.9769, and 0.9280, respectively. The proposed model is expected to contribute to precision medicine by potentially saving examination-related time and labor and reducing disagreements among pathologists.

## 1. Introduction

Gastric cancer is the most common type of cancer, accounting for 12% of all cancer cases in Korea according to data from the National Cancer Center in 2018 [1]. In 2020, more than 1 million (1,089,103) new cases of gastric cancer were estimated worldwide, resulting in 768,793 deaths [2]. Lymph node metastasis is the most significant prognostic factor for patients with gastric cancer, and the presence of lymphovascular invasion (LVI) is the most significant risk factor for lymph node metastasis [3,4,5,6]. LVI is defined as the invasion of vessel walls by tumor cells and/or the presence of tumor emboli within an endothelial-lined space [7]. Predictive value and prevalence of LVI are highly dependent on the type of cancer, and the presence of LVI is a recognized prognostic factor in a variety of solid malignancies, including breast cancer, urothelial carcinoma, and colorectal cancer [8]. Since the proclamation of LVI as an important factor in the prognosis of gastric cancer by Talamonti et al. [9], the American Joint Committee on Cancer has recommended the evaluation of LVI [10]. According to the current Japanese guidelines, LVI in gastric cancer is not clinically useful information except for predicting the possibility of curative endoscopic resection. LVI is the most significant risk factor associated with lymph node metastases in individuals with early gastric cancer [6,11,12,13]. The rate of lymph node metastasis observed in patients exhibiting LVI (25.7–32.1%) was much higher compared to that in those without LVI (1.5–2.3%) [6,11,13,14]. In addition, Fusikawa et al. showed that a significant difference was observed between the values of 79.8% in the LVI(–) group and 67.2% in the LVI(+) group in advanced cancer [7]. The five-year survival rate of advanced cancers with nodal metastases is 76.7% in the LVI(–) group and 60.9% in the LVI(+) group [7]. Therefore, LVI is an independent prognostic marker in gastric cancer and tends to worsen the prognosis, particularly in cases of advanced malignancy with lymph node metastasis.

The recognition of lymphatic tumor emboli in microscopic sections is dependent on the pathologist [15]. There is potential for significant inter-observer variations in the diagnosis of LVI amongst pathologists [16]. Inter-observer disagreement can be expected in the diagnosis of LVI as retraction artifacts that isolate tumor aggregates can be caused by tissue shrinkage during fixation, which are easily confused with true tumor emboli during routine examination of hematoxylin and eosin (H&E) stained sections [17,18]. Tumors may be artefactually displaced into vessels during specimen cut up or processing [19]. For instance, Gilchrist et al. noted that when three surgical pathologists were told to assess for LVI in a pT1-2 N0 M0 histological mastectomy case, all three concurred in only 12 of 35 breast cancer cases [15,16]. Several attempts have been made to overcome these limitations. The monoclonal D2-40 antibody can selectively detect lymphatic vessels as it is expressed in the lymphatic endothelium but not in blood vessels, and D2-40 staining is reportedly more sensitive than H&E staining for detecting lymphatic invasion (LI) [17,20,21]. Elastin staining may also be used for a clearer recognition of blood vessels as it identifies the elastic fibers of blood vessels [22,23,24,25]. Inter-observer agreement in the diagnoses of LVI was improved by adding ancillary D2-40 and elastin staining, regardless of the experience of the pathologists [4]. However, the assessment of LVI by pathologists is inherently limited owing to human errors. Examining large areas of tumors for LVI is time-consuming and challenging because the foci of LVI can be small and subjective. Nonetheless, the presence of LVI can have a marked impact on disease management, and the identification of a genuine single focus is sufficient to label a case as LVI(+). This automated identification of possible LVI(−)indicating lesions may have significant clinical utility [19]. 

Digital pathology defines the creation of whole-slide images (WSI) from a histology slide that can be viewed on a screen to form a diagnostic report [26]. Traditionally, histological diagnosis and pathological staging by pathologists have been evaluated using glass slides and microscopes [26]. Digital pathology is now increasingly being implemented in laboratories around the world, and digital support management is seen as a key component of health service planning aimed at improving efficiency, network operation, and quality [26]. There is great potential for using artificial intelligence (AI) to assist pathologists and derive new biological insights into disease biology, even in areas imperceptible to human observers [27]. However, the majority of AI medical devices that have received FDA approval and have been introduced to the market thus far are primarily focused on radiology. In contrast, only a limited number of devices have been approved for use in the field of pathology [28]. Moreover, it is important to explore the potential of these AI technologies as many pathology departments do not have enough pathologists.

AI algorithms that utilize convolutional neural networks (CNNs) for image analysis have already shown significant promise in the pathological evaluation of various solid tumors, including prostate cancer screening in prostate biopsies [29,30], leading to new evaluations of clinical outcomes, providing [31,32] or predicting the presence of mutations [33] or molecular subtypes [34] in H&E-stained sections. The usefulness of these algorithms in identifying small regions of prognostic significance in digital WSI has previously been demonstrated in the context of identifying metastatic breast cancer within lymph nodes [35,36]. In addition, the AI model can automatically find LVI in the WSI of testicular cancer [19]. AI model can identify LVI foci better than a human expert (recall score: 0.68 vs. 0.56). 

**Figure 1:**
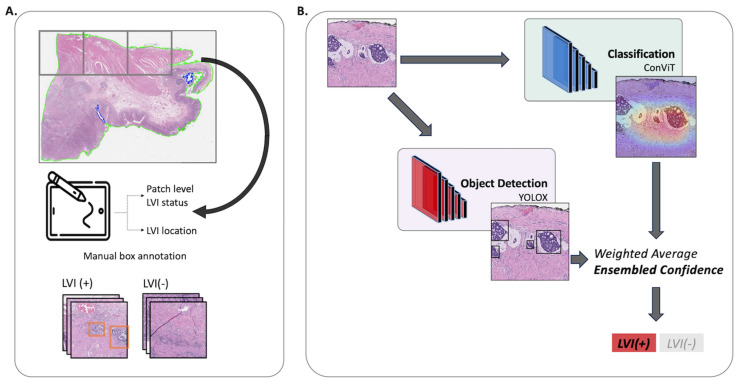
Schematic of the LVI Net. Panel (**A**) portrays the preprocessing step and annotations, while Panel (**B**) illustrates the workflow of the LVI Net. The patch image is input into both the classification and detection models. Subsequently, the prediction outcomes from these models conducted weighted averaging, resulting in the computation of the final confidence level (referred to as the ensemble confidence). This ensemble confidence is then utilized to predict the ultimate diagnosis of LVI(+) or LVI(−).

In this study, we developed an algorithm to identify LVI foci related to the prognosis of gastric cancer. The image classification and detection models were trained and validated at both the patch and WSI levels. The ensemble approach was used to combine the predictions of these sub-models to improve the overall performance of the model. The sub-models were trained on a dataset of WSI of gastric cancer, with annotations of vascular and lymphatic vascular invasion. A conceptual diagram of the LVI prediction model is shown in Figure 1.

## 2. Methods

### 2.1. Patients and Tumor Samples

Gastric adenocarcinoma slides were obtained from 88 patients who underwent endoscopic submucosal dissection, subtotal gastrectomy, or total gastrectomy at the Chonnam National University Hwasun Hospital from 2018 to 2021. The availability of adequate tissue and the histological diagnosis of gastric cancer were the inclusion criteria. One hundred WSI were collected from these patient samples. Clinical information was collected from the electronic medical records maintained in the electronic database of the hospital. This study was approved by the Institutional Review Board (IRB) of the Chonnam National University Hwasun Hospital (CNUHH-2021-197) and conducted in accordance with the Declaration of Helsinki. Informed consent from patients was waived with IRB approval.

### 2.2. Datasets

**Figure 2:**
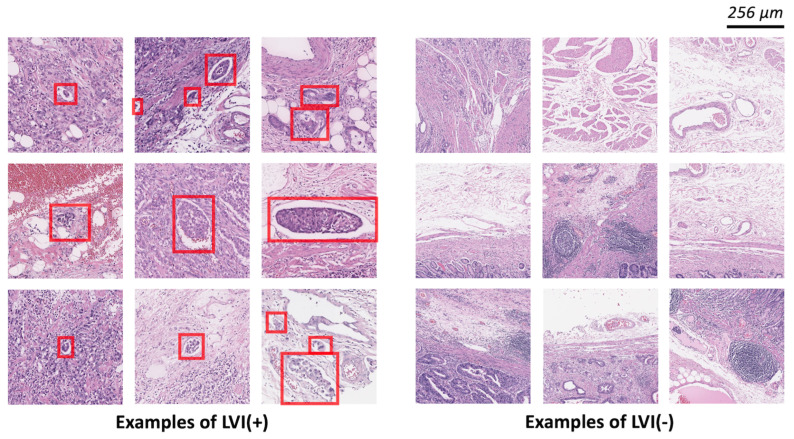
Example patch images of LVI(+) and LVI(−). The left panel displays a patch associated with LVI(+) classification, while the right panel represents LVI(−). LVI foci refer to tumors located within identifiable white, rounded structures that align anatomically with blood vessels and lymph nodes. A patch is classified as positive if it contains one or more regions indicating the presence of LVI. The LVI areas are marked as red boxes.

The slides were scanned using a Leica-Aperio GT450 Scanner (Leica Biosystems) using an 40× objective. Using QuPath 0.3.0 tools, the LVI(+) regions were annotated by two board-certified pathologists. The examples of LVI(+) and LVI(−) are depicted in Figure 2. We performed CD34 and D2-40 immunohistochemical staining on all slides to confirm LVI(+) foci and to increase the accuracy of marking LVI(+) foci. For training, validation, and test splitting, we randomly selected WSI with a 6:2:2 ratio. We patchified WSIs using conventional digital pathology image analysis (Figure 1, preprocessing panel). LVI(+) foci were generated based on LVI(+) annotations. The sliding windowing approach generated LVI(−) patches from the remaining WSI. Without any overlap, we visited all WSI regions that did not include LVI(+) foci. To handle class imbalances and remove redundancy in LVI(−) patches, one-third was sampled from all LVI(−) patches. The LVI(+) and LVI(−) patches were generated at 20×-level (0.5 µm/pixel) with 512 × 512 pixels. 

To conduct external validation, we utilized a publicly accessible classification dataset that contained patch images pertaining to lymphatic invasion [37]. Comprising 48 WSIs sourced from 27 patients, this external validation dataset comprised 302 positive instances and 671 negative instances. The patch images were captured at a 5×-level magnification (2 µm/pixel) with dimensions of 512 × 512 pixels. Notably, this external validation dataset was acquired using a distinct scanner (Leica-Aperio AT2) and originated from a different hospital setting.

### 2.3. Model Development

We fine-tuned the image classification and detection models to identify the LVI foci in a given patch image. The following analysis was conducted using Python 3.8, Pytorch 1.13.1, and a single A100 GPU. 

### 2.4. Classification Models

We defined the classification problem as a binary classification. The ResNet 50 [38], EfficeientNet B3 [39], and ConViT (Small) [40] models were fine-tuned on the LVI datasets. The parameters of the selected image classification models ranged from 20 to 30 M. In an empirical study, we found that large parameters converged into overfitting because of our limited dataset volume. We utilized ImageNet [41] pretrained weights with entire layers that can be updated by considering the modality gap between a conventional RGB and digital pathology images. Image augmentations were applied, including affine transform, elastic transform, blurring, brightness, and color jittering. Balanced weight-sampling methods were applied during training to alleviate data imbalance. The image classification models were trained using the Adam optimizer (learning rate: 1 × 10^−4^), cosine annealing learning rate scheduler, and automated mixed precision. 

### 2.5. Detection Models

The detection model was utilized to classify and localize the desired object in the entire image simultaneously. A regression operation was applied to localize the object using a bounding box. We utilized a one-stage object detection model called the YOLO model [42]. YOLO detection uses the concept of an anchor box. The anchor box has a predefined shape and ratio of the bounding box that is utilized in the bounding box location prediction. For example, human objects commonly exhibited square shapes with long heights and short widths. In contrast, the dog objects had square shapes with short heights and log widths. Anchor-based methods have been actively utilized to ease the prediction performance. However, in terms of LVI, the shape of LVI was arbitrary; several LVI foci assumed a square shape, and the others assumed a rectangular shape with variants of size. To compare the impact of the anchor box assumption on LVI foci detection, we trained both an anchor box assumption-based detection model (YOLO v3) [43] and detection model without the anchor box assumption (YOLOX) [44]. To match the number of parameters, the medium size of YOLOX was selected. The hyperparameters and data augmentations followed the recommendations of each framework. The detection model could detect as many LVI(+) regions as possible. Therefore, unlike a classification model, a single-patch image can have multiple prediction confidence scores. To aggregate multiple confidence scores, we computed the augmented confidence score of each patch image using the maximum operator.

### 2.6. Ensemble Model

The ensembled confidence score ($C_{ens}$) is calculated as the weighted average of the confidence score of the classification model ($C_{clf}$) and the augmented confidence score of the detection model ($C_{det}$), according to Equation (1):

(1) $$C_{ens}=\frac{\left(w_{clf}{\;\times \;C}_{clf}+w_{det}\;{\times \;C}_{det}\right)}{2},$$

where $w_{clf}$ and $w_{det}$ denote weighted factors of classification and detection models, respectively. Considering the performances of each model, we empirically set the $w_{clf}$ to 1.0 and $w_{det}$ to 1.0, respectively. The ensembled confidence score was treated as a final confidence score.

### 2.7. Evaluation Metrics

Generally, to evaluate the classification performance, the true positive (TP), false positive (FP), false negative (FN), and true negative (TN) are computed by comparing the prediction confidence that a model returns and the ground truth. Furthermore, the TP, FP, FN, and TN, accuracy score, recall (sensitivity), precision (positive predicted value, PPV), F1 score, AUROC, AUPRC are obtained. The detection performance was evaluated based on the intersection over union (IOU) of the bounding box predicted by the model and ground truth bounding box. With the IOU threshold, we could determine whether the model prediction was true or false. Using the precision and recall scores, we can summarize the detection performance as an average precision (AP) score [45]. The AP_50_ score corresponded to the AP score at the IOU threshold of 50%. The classification performance of the detection model was computed based on the augmented confidence score that aggregated multiple prediction outputs.

## 3. Results

### 3.1. Patient Characteristics

All the patients were LVI(+). The mean age of the patients was 69.6 years (±10.2), and the majority were men (73.0%) (Table 1). Poorly differentiated tumors comprised 46.0% of the cases. Despite being LVI(+), 10 patients (18.2%) did not exhibit LNM. The number of lymph node involvement was 12.0 (±13.7). Perineural invasion was observed in 39 (61.9%) patients. The clinicopathological features of the cases are summarized in Table 1.

### 3.2. Patch-Level Analysis

**Figure 3:**
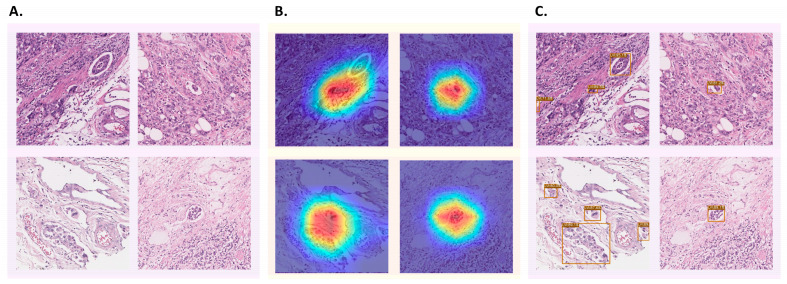
The output of classification and detection model. The summarization of the classification and detection results for the same patch image is presented. Panel (**A**) displays the original image, while panels (**B**,**C**) showcase the classification and detection results, respectively. The heatmap generated using Grad-CAM highlights the areas of focus by the classification model, with red areas indicating greater attention. This visual representation indicates that the classification model exhibits a relatively focused perspective. Conversely, the detection model predicts the object’s location by enclosing it within a bounding box and provides the confidence level for each prediction. It is evident that the detection model successfully identifies various dispersed regions within the image.

With WSI-level splitting, each WSI was randomly allocated as a training, valid, or test dataset. Each WSI image had a different prognosis for LVI. Therefore, the number of LVI foci and patch images was heterogeneous. The dataset configurations are presented in Table 2. The patch-level analysis was components: classification, detection, and an ensemble of both classification and detection. Figure 3 illustrated the example outputs of ground truths, classification focused areas, and detection outputs. 

### 3.3. Patch-Level Analysis: Classification Models

The patch classification results were outstanding for all classification models without any considerable performance gap. The ConViT model showed the best performance in terms of both the area under the receiver operating characteristics (AUROC) and area under the precision-recall curve (AUPRC) in the classification models (AUROC: 0.9796; AUPRC: 0.9648). The accuracy, precision, recall score, and F1 score were computed with a confidence score threshold of 0.5.

### 3.4. Patch-Level Analysis: Detection Models

In detection, the YOLOX model outperformed the YOLO v3 model in both detection (AP_50_) and classification metrics. The AP_50_ of YOLOX and YOLO v3 were 0.55 and 0.66, respectively. The AUROC and AUPRC values of YOLOX were higher than those for YOLO v3 (0.9666 vs. 0.9702 for the AUROC and 0.9423 vs. 0.9302 for the AUPRC). However, the AUROC and AUPRC of YOLOX computed based on the augmented patch-level confidence score were slightly lower (AUROC: −0.0094; AUPRC: −0.0225) than those of the ConViT classification model. In the detection models, the accuracy, precision, recall score, and F1 score were computed with an augmented patch-level confidence score threshold of 0.7. The threshold was adjusted to be stricter than the value utilized in the image classification model to mitigate the heavy false positives that could occur during detection. 

### 3.5. Patch-Level Analysis: Ensemble Model

Notably, the YOLOX model exhibited an outstanding F1 score (+0.0039 points compared with that of ConViT) in all benchmark models. Considering the AUROC, AUPRC, and F1 scores, we attempted to mix the best-performing models in an ensemble approach. With simple averaging of the patch-level confidence scores, the ensemble model showed the best AUROC, AUPRC, and F1 scores of 0.9880, 0.9769, and 0.9280, respectively. The performances are summarized in Table 3. 

### 3.6. WSI-Level Analysis

**Figure 4:**
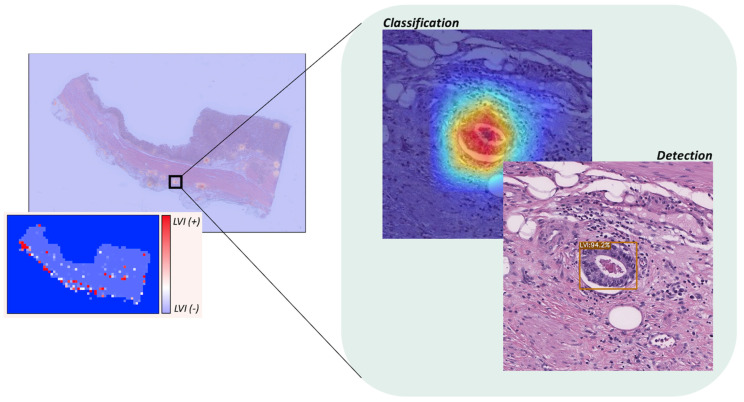
The example WSI-level analysis. A WSI-level analysis can be visualized by combining the results of patch-level analysis. Additionally, it can be illustrated as a WSI-level heatmap, which utilizes the spatial information of the patch images. In the heatmap representation, red points indicate regions that exhibit high confidence in being classified as LVI(+) cases, whereas blue points indicate regions with high confidence in being classified as LVI(−) cases. The image on the right showcases a magnified view of the area identified as LVI(+), presenting the respective judgments made by both the classification model and the detection model.

The WSI consists of multiple patch images, allowing for aggregation of these patches at the WSI level. The conceptual diagram of WSI-level analysis is shown in Figure 4. Each patch prediction result was aggregated at the WSI-level, and the WSI-level prediction result was aggregated once more in the entire test dataset. The WSI-level prediction performance is summarized in Table 4. The performance was consistent with the results of the patch-level prediction (Table 3). We adjusted the threshold such that the positive and negative could be determined as the medium points (0.5); however, this threshold could be rescaled depending on the interests of the researcher. In our dataset, WSIs generally included multiple LVI regions. Therefore, we concluded that the benefit of reducing false positives was more significant. If the LVI region is small, such as in patients with early-stage cancer, a strategy can be adopted to reduce false negatives by lowering the threshold. 

### 3.7. External Validation

To measure the efficacy of the ensemble approach, we conducted an external validation using a preexisting dataset. Employing this classification dataset facilitated the application of our model to ascertain positive or negative LVIs [37]. The ensemble model demonstrated superior performance compared to both classification and object detection models (Table 5). Specifically, the AUROC of the ensemble model exhibited improvements of 0.025 (2.8%) and 0.052 (5.9%) in contrast to the classification and detection models, respectively. Furthermore, the AUPRC of the ensemble model saw enhancements of 0.044 (5.1%) and 0.081 (9.8%), respectively. Analogous to the internal validation dataset, the ensemble model exhibited robustness when compared to the classification and detection-only models.

## 4. Discussion

In this study, we present a deep-learning model for predicting gastric LVI from the patch images from WSI. Two models were developed: image classification and detection. The ConViT (classification) and YOLOX (detection) models showed comparable performances. The final ensemble model showed outstanding performance in predicting gastric LVI. 

In a previous study, Ghosh et al. demonstrated that a deep-learning model could predict LVI foci in testicular LVI [36]. They applied the semantic segmentation-based model (DeeplabV3) [46] to predict the mask of LVI foci; however, the number of LVI(+) foci to train and evaluate a semantic segmentation model were small. Therefore, the model performance can be further improved. With few samples of LVI foci included in the test dataset (34 foci), it could be difficult to determine the generalized performance of LVI prediction. 

One of the primary tasks of digital pathology is the detection of mitosis, which often employs a two-stage framework comprising object detection and classification [47]. This approach is preferred due to the small size of mitotic objects, which makes the model predictions highly susceptible to false positives and false negatives. Initially, candidate regions are identified through object detection, and subsequently refined using classification techniques. While the sequential application of this two-stage framework may not pose significant challenges in studies based on limited benchmark datasets, it can prove time-consuming in typical medical scenarios. Therefore, to address this issue, we propose an ensemble approach that combines the advantages of the two-stage model while enabling parallel processing.

In our experimental setting, the classification model ConViT exhibited an outstanding performance among the candidate classification models. The ConViT model attempted to fuse the outstanding performance of transformer-based architectures with the advantages of CNN. The ability of the transformer to focus on global information and the ability of CNNs to focus on local patterns boosted the prediction performance. The LVI foci had heterogeneous shape and size characteristics. In addition, it is essential to determine whether the LVI is located in the lymph node site or blood vessels. The most common false positives occurred in detachment artifacts owing to the failure to interpret peripheral contexts.

The detection model also showed comparable performance in detecting LVI foci. The anchor-free assumption-based model YOLOX was more appropriate because of the varying sizes and shapes of the LVI foci. The YOLOX model exhibited a comparable performance with regard to the AUROC and AUPRC than the ConViT model. However, it exhibited a slightly better performance with regard to the F1 score. The ensemble model exhibited improved AUROC, AUPRC, and F1 scores compared with the classification and detection-only model (improved gain: 0.0084, AUROC; 0.012, AUPRC; 0.022, F1 score). Additionally, the improvement of the ensemble model was also found in the external validation (AUROC: 2.8%; AUPRC: 5.1%). 

Our model predicted LVI foci in WSI; in other words, it identified whether LVI foci existed. However, LVI is essentially a histological finding that suggests the possibility of metastasis to the lymph nodes. Previous studies have reported models to predict LNM from pathological slide images of solid tumors, such as breast, colorectal, bladder, and prostate cancers. Although LNM is one of the most important prognostic factors, a model for predicting LNM in gastric cancer has not yet been reported. Wang et al. reported a model for predicting the prognosis of gastric cancer using the histopathology of resected lymph nodes; however, this was not a model for predicting metastasis to the lymph nodes. This algorithm, which detects LVI(+) foci, is expected to significantly help pathologists at the actual reading site, However, predicting LVI(+) in the clinical field is not sufficient to predict the prognosis of a patient. It is necessary to conduct additional studies on the association of the LVI(+) foci identified by this algorithm with the number of lymph node metastases and patient survival prognosis, and thus, further investigation into this is anticipated.

In addition, semi-supervised and active learning pipelines for generating LVI focal labeling more easily need to be further developed. Our YOLOX model can predict the LVI foci using a bounding box. Therefore, we can assume that the prediction results of YOLOX are newly annotated LVI foci in the other datasets. With the supervision of human experts who reject or accept newly annotated LVI foci (active learning), the labeled dataset expands rapidly. Additionally, in this study, we hypothesized that detection would be sufficient to predict LVI foci. However, a previous study utilized semantic-segmentation-based modeling for testicular LVI foci detection. LVI foci share similar patterns despite differences in organs, such as tumors surrounded by blood vessels or lymph nodes. Therefore, in the future, we aim to expand our work to compare semantic segmentation, object detection, and classification models to predict the LVI foci. 

Our study has several limitations. First, a number of LVI(+) foci imbalances may exist for each slide. This data imbalance problem may cause distortion in the learning process. We applied WSI-level data splitting to resolve the LVI(+) foci imbalance problem. The best option for data splitting involves splitting the patient-level data. However, we encountered varying LVI(+) foci depending on the patient status. Furthermore, of the multiple sections of slides that may be present in a single gastric cancer tissue, we selected no more than five slides from the same patient. Therefore, patient-level data splitting can be coupled with a heavy class imbalance that is harmful to supervised learning procedures. To mitigate this issue, we alternately selected WSI-level splitting. Second, LVI(+) foci always contain the possibility of false positives or negatives. To reduce false-positive or false-negative foci marks at the annotation step, we confirmed CD34 and D2-40 immunohistochemical staining on all slides. In addition, LVI(+) confirmation was performed by two pathologists. However, annotation marking for foci may be missed because LVI(+) is a relatively small lesion within the WSI. This results in missed marking annotations for some LVI(+) foci and marked LVI(+) foci for some artifacts. Similarly, when the trained algorithmic model predicts LVI(+) positive foci, it may be a false positive. To discriminate false positives, all areas predicted to be LVI(+) positive foci were individually checked by two pathologists. Through this process, we were able to improve the accuracy of the model in predicting LVI(+) foci. Spatial heterogeneity is a crucial factor that must be taken into account in studies on artificial intelligence learning in digital pathology. Stomach cancer is specifically recognized as a type of cancerous tissue that exhibits significant and pronounced spatial heterogeneity within the tissue. Nevertheless, spatial heterogeneity was not a significant factor that needed to be taken into account for this project. LVI is histopathologically defined by the presence of tumor emboli within lymphatic/vascular channels and exhibits morphological features that are rather homogeneous. For instance, the presence of LVI is not exclusive to stomach cancer but is also observed in various other forms of cancer. These findings indicate that the scope of this research extends beyond stomach cancer and has potential for further application to other types of cancer.

## 5. Conclusions

This research presents an ensemble deep-learning model for detecting vascular and lymphatic vascular invasion in WSI of histopathology of gastric cancer. The ensemble deep-learning model has been demonstrated as more robust and accurate than single models, and it can be used as a valuable tool for pathologists in diagnosing gastric cancer and may help improve the accuracy of diagnosis and prognosis of the disease. This approach can be considered an alternative to traditional methods and as a step toward computer-aided diagnotic systems in histopathology.

## Figures and Tables

**Table 1:** Baseline characteristics of the study population.

Variable	Total (N = 63)
Age ^1^	69.6 (10.2)
Sex ^2^	
Male	46 (73.0%)
Female	17 (27.0%)
Lauren Classification ^2^	
Intestinal	36 (57.1%)
Diffuse	12 (19.0%)
Mixed	15 (23.8%)
Grade ^2^	
Well differentiated	3 (4.8%)
Moderately differentiated	31 (49.2%)
Poorly differentiated	29 (46.0%)
T Staging ^2^	
pT1a	2 (3.2%)
pT1b	17 (27.0%)
pT2	5 (7.9%)
pT3	13 (20.6%)
pT4a	23 (36.5%)
pT4b	3 (4.8%)
N Staging ^2^	
pN0	10 (18.2%)
pN1	6 (10.9%)
pN2	13 (23.6%)
pN3a	8 (14.5%)
pN3b	18 (32.7%)
LN Involvement ^1^	12.0 (13.7)
Perineural Invasion ^2^	
Present	39 (61.9%)
Not identified	24 (38.1%)
IHC Expression of C-erb B2 ^2^	
0	36 (57.1%)
1+	11 (17.5%)
2+	6 (9.5%)
3+	7 (11.1%)
Not available	3 (4.8%)

LN, Lymph Node; IHC, Immunohistochemistry. ^1^ Mean (S.D.); ^2^ Number of items (Percentage).

**Table 2:** Dataset configuration.

	# of WSI	# of Positive Per WSI ^1^	# of Negative Per WSI ^1^
Train set	64	68.77 (90.04)	159.23 (87.54)
Valid set	16	28.50 (25.61)	161.12 (60.69)
Test set	20	105.3 (91.73)	201.4 (102.48)

WSI, Whole slide image; ^1^ Mean (S.D.).

**Table 3:** Performance of trained model using the patch images.

Method	Model	AUROC	AUPRC	Accuracy	F1 Score
Classification	ResNet50	0.9762 (0.9726–0.9798)	0.9593 (0.9447–0.9739)	0.9319 (0.9254–0.9384)	0.8992 (0.8895–0.9089)
	EfficientNetB3	0.9731 (0.9693–0.9769)	0.9551 (0.935–0.9752)	0.9281 (0.9217–0.9345)	0.8929 (0.8827–0.9031)
	ConViT	0.9796 (0.9765–0.9827)	0.9648 (0.9592–0.9704)	0.9348 (0.9288–0.9408)	0.9025 (0.8935–0.9115)
Detection	YOLOv3	0.9666 (0.9623–0.9709)	0.9302 (0.9203–0.9401)	0.927 (0.9196–0.9344)	0.8977 (0.8868–0.9086)
	YOLOX	0.9702 (0.9648–0.9756)	0.9423 (0.9323–0.9523)	0.9353 (0.9278–0.9428)	0.9064 (0.8962–0.9166)
Ensemble		0.988 (0.9852–0.9908)	0.9769 (0.9717–0.9821)	0.9514 (0.9459–0.9569)	0.928 (0.9198–0.9362)

Mean (95% confidence interval).

**Table 4:** Performance of trained model using the whole slide images.

Method	True Negative Rate ^1^	False Positive Rate ^1^	False Negative Rate ^1^	True Positive Rate ^1^
ConViT	96.63 (0.03)	3.37 (0.03)	11.88 (0.09)	88.12 (0.09)
YOLOX	94.99 (0.03)	5.01 (0.03)	10.88 (0.12)	89.12 (0.12)
Ensemble	97.56 (0.02)	2.44 (0.02)	10.21 (0.07)	89.79 (0.07)

^1^ Mean (S.D.).

**Table 5:** Performance of trained model using the external validation dataset.

Method	Model	AUROC	AUPRC	Accuracy	F1 Score
Classification	ConViT	0.9184 (0.8975–0.9393)	0.869 (0.8338–0.9041)	0.8674 (0.8465–0.8883)	0.7896 (0.7543–0.8248)
Detection	YOLOX	0.8915 (0.8638–0.9192)	0.8319 (0.7876–0.8763)	0.8592 (0.8364–0.882)	0.7934 (0.7577–0.8291)
Ensemble		0.9438 (0.9258–0.9619)	0.9132 (0.8875–0.939)	0.8983 (0.879–0.9175)	0.8358 (0.8035–0.8681)

Mean (95% confidence interval).

## Data Availability

Data cannot be shared publicly because of sensitive personal medical information. The datasets generated and/or analyzed during the current study are available from the corresponding author upon reasonable request. The external validation dataset is available at https://zenodo.org/records/10020633 (accessed on 3 January 2024). The source code of this project available at https://github.com/jonghyunlee1993/LVINet (accessed on 9 January 2024).

## References

[1] Hong S., Won Y.J., Lee J.J., Jung K.W., Kong H.J., Im J.S., Seo H.G., The Community of Population-Based Regional Cancer Registries (2021). Cancer statistics in Korea: Incidence, mortality, survival, and prevalence in 2018. Cancer Res. Treat..

[2] Lordick F., Carneiro F., Cascinu S., Fleitas T., Haustermans K., Piessen G., Vogel A., Smyth E.C. (2022). Gastric cancer: ESMO clinical practice guideline for diagnosis, treatment and follow-up. Ann. Oncol..

[3] Ferlay J., Ervik M., Lam F., Colombet M., Mery L., Piñeros M. Global Cancer Observatory: Cancer Today. International Agency for Research on Cancer. https://gco.iarc.fr/today.

[4] Takada K., Yoshida M., Aizawa D., Sato J., Ono H., Sugino T. (2020). Lymphovascular invasion in early gastric cancer: Impact of ancillary D2-40 and elastin staining on interobserver agreement. Histopathology.

[5] Nitti D., Marchet A., Olivieri M., Ambros A., Mencarelli R., Belluco C., Lise M. (2003). Ratio between metastatic and examined lymph nodes is an independent prognostic factor after D2 resection for gastric cancer: Analysis of a large European monoinstitutional experience. Ann. Surg. Oncol..

[6] Sekiguchi M., Oda I., Taniguchi H., Suzuki H., Morita S., Fukagawa T., Sekine S., Kushima R., Katai H. (2016). Risk stratification and predictive risk-scoring model for lymph node metastasis in early gastric cancer. J. Gastroenterol..

[7] Gotoda T., Yanagisawa A., Sasako M., Ono H., Nakanishi Y., Shimoda T., Kato Y. (2000). Incidence of lymph node metastasis from early gastric cancer: Estimation with a large number of cases at two large centers. Gastric Cancer.

[8] Fujikawa H., Koumori K., Watanabe H., Kano K., Shimoda Y., Aoyama T., Yamada T., Hiroshi T., Yamamoto N., Cho H. (2020). The clinical significance of lymphovascular invasion in gastric cancer. In Vivo.

[9] Song Y.J., Shin S.H., Cho J.S., Park M.H., Yoon J.H., Jegal Y.J. (2011). The role of lymphovascular invasion as a prognostic factor in patients with lymph node-positive operable invasive breast cancer. J. Breast Cancer.

[10] Talamonti M.S., Kim S.P., Yao K.A., Wayne J.D., Feinglass J., Bennett C.L., Rao S. (2003). Surgical outcomes of patients with gastric carcinoma: The importance of primary tumor location and microvessel invasion. Surgery.

[11] Amin M.B., Greene F.L., Edge S.B., Compton C.C., Gershenwald J.E., Brookland R.K., Meyer L., Gress D.M., Byrd D.R., Winchester D.P. (2017). The eighth edition AJCC cancer staging manual: Continuing to build a bridge from a population-based to a more “personalized” approach to cancer staging. CA Cancer J. Clin..

[12] Kim Y.I., Kook M.C., Choi J.E., Lee J.Y., Kim C.G., Eom B.W., Yoon H.M., Ryu K.W., Kim Y.W., Choi I.J. (2020). Evaluation of submucosal or lymphovascular invasion detection rates in early gastric cancer based on pathology section interval. J. Gastric Cancer.

[13] Kwee R.M., Kwee T.C. (2008). Predicting lymph node status in early gastric cancer. Gastric Cancer.

[14] Kim H., Kim J.H., Park J.C., Lee Y.C., Noh S.H., Kim H. (2011). Lymphovascular invasion is an important predictor of lymph node metastasis in endoscopically resected early gastric cancers. Oncol. Rep..

[15] Lee S.Y., Yoshida N., Dohi O., Lee S.P., Ichikawa D., Kim J.H., Sung I.K., Park H.S., Otsuji E., Itoh Y. (2017). Differences in prevalence of lymphovascular invasion among early gastric cancers between Korea and Japan. Gut Liver.

[16] Zaorsky N.G., Patil N., Freedman G.M., Tuluc M. (2012). Differentiating lymphovascular invasion from retraction artifact on histological specimen of breast carcinoma and their implications on prognosis. J. Breast Cancer.

[17] Gilchrist K.W., Gould V.E., Hirschl S., Imbriglia J.E., Patchefsky A.S., Penner D.W., Pickren J., Schwartz I.S., Wheeler J.E., Barnes J.M. (1982). Interobserver variation in the identification of breast carcinoma in intramammary lymphatics. Hum. Pathol..

[18] Gresta L.T., Rodrigues-Junior I.A., de Castro L.P., Cassali G.D., Cabral M.M. (2013). Assessment of vascular invasion in gastric cancer: A comparative study. World J. Gastroenterol..

[19] Ghosh A., Sirinukunwattana K., Khalid Alham N., Browning L., Colling R., Protheroe A., Protheroe E., Jones S., Aberdeen A., Rittscher J. (2021). The potential of artificial intelligence to detect lymphovascular invasion in testicular cancer. Cancers.

[20] Yonemura Y., Endou Y., Tabachi K., Kawamura T., Yun H.Y., Kameya T., Hayashi I., Bandou E., Sasaki T., Miura M. (2006). Evaluation of lymphatic invasion in primary gastric cancer by a new monoclonal antibody, D2-40. Hum. Pathol..

[21] Arigami T., Natsugoe S., Uenosono Y., Arima H., Mataki Y., Ehi K., Yanagida S., Ishigami S., Hokita S., Aikou T. (2005). Lymphatic invasion using D2-40 monoclonal antibody and its relationship to lymph node micrometastasis in pN0 gastric cancer. Br. J. Cancer.

[22] Sako A., Kitayama J., Ishikawa M., Yamashita H., Nagawa H. (2006). Impact of immunohistochemically identified lymphatic invasion on nodal metastasis in early gastric cancer. Gastric Cancer.

[23] Araki I., Hosoda K., Yamashita K., Katada N., Sakuramoto S., Moriya H., Mieno H., Ema A., Kikuchi S., Mikami T. (2015). Prognostic impact of venous invasion in stage IB node-negative gastric cancer. Gastric Cancer.

[24] Harris E.I., Lewin D.N., Wang H.L., Lauwers G.Y., Srivastava A., Shyr Y., Shakhtour B., Revetta F., Washington M.K. (2008). Lymphovascular invasion in colorectal cancer: An interobserver variability study. Am. J. Surg. Pathol..

[25] Kirsch R., Messenger D.E., Riddell R.H., Pollett A., Cook M., Al-Haddad S., Streutker C.J., Divaris D.X., Pandit R., Newell K.J. (2013). Venous invasion in colorectal cancer impact of an elastin stain on detection and interobserver agreement among gastrointestinal and nongastrointestinal pathologists. Am. J. Surg. Pathol..

[26] Nam S., Chong Y., Jung C.K., Kwak T.Y., Lee J.Y., Park J., Rho M.J., Go H. (2020). Introduction to digital pathology and computer-aided pathology. J. Pathol. Transl. Med..

[27] Ahmad Z., Rahim S., Zubair M., Abdul-Ghafar J. (2021). Artificial intelligence (AI) in medicine, current applications and future role with special emphasis on its potential and promise in pathology: Present and future impact, obstacles including costs and acceptance among pathologists, practical and philosophical considerations. A comprehensive review. Diagn. Pathol..

[28] Joshi G., Jain A., Araveeti S.R., Adhikari S., Garg H., Bhandari M. (2022). FDA approved Artificial Intelligence and Machine Learning (AI/ML)-Enabled Medical Devices: An updated landscape. medRxiv.

[29] Pantanowitz L., Quiroga-Garza G.M., Bien L., Heled R., Laifenfeld D., Linhart C., Sandbank J., Albrecht Shach A., Shalev V., Vecsler M. (2020). An artificial intelligence algorithm for prostate cancer diagnosis in whole slide images of core needle biopsies: A blinded clinical validation and deployment study. Lancet Digit. Health.

[30] Campanella G., Hanna M.G., Geneslaw L., Miraflor A., Werneck Krauss Silva V., Busam K.J., Brogi E., Reuter V.E., Klimstra D.S., Fuchs T.J. (2019). Clinical-grade computational pathology using weakly supervised deep learning on whole slide images. Nat. Med..

[31] Turkki R., Byckhov D., Lundin M., Isola J., Nordling S., Kovanen P.E., Verrill C., von Smitten K., Joensuu H., Lundin J. (2019). Breast cancer outcome prediction with tumour tissue images and machine learning. Breast Cancer Res. Treat..

[32] Bychkov D., Linder N., Turkki R., Nordling S., Kovanen P.E., Verrill C., Walliander M., Lundin M., Haglund C., Lundin J. (2018). Deep learning based tissue analysis predicts outcome in colorectal cancer. Sci. Rep..

[33] Wang S., Shi J., Ye Z., Dong D., Yu D., Zhou M., Liu Y., Gevaert O., Wang K., Zhu Y. (2019). Predicting EGFR mutation status in lung adenocarcinoma on computed tomography image using deep learning. Eur. Respir. J..

[34] Hinata M., Ushiku T. (2021). Detecting immunotherapy-sensitive subtype in gastric cancer using histologic image-based deep learning. Sci. Rep..

[35] Bejnordi B.E., Veta M., van Diest P.J., van Ginneken B., Karssemeijer N., Litjens G., van der Laak J.A.W.M., Consortium C. (2017). Diagnostic assessment of deep learning algorithms for detection of lymph node metastases in women with breast cancer. JAMA.

[36] Steiner D.F., MacDonald R., Liu Y., Truszkowski P., Hipp J.D., Gammage C., Thng F., Peng L., Stumpe M.C. (2018). Impact of deep learning assistance on the histopathologic review of lymph nodes for metastatic breast cancer. Am. J. Surg. Pathol..

[37] Lee J., Ahn S., Kim H.S., An J., Sim J. (2024). A robust model training strategy using hard negative mining in a weakly labeled dataset for lymphatic invasion in gastric cancer. J. Pathol. Clin. Res..

[38] He K., Zhang X., Ren S., Sun J. Deep residual learning for image recognition. Proceedings of the IEEE Conference on Computer Vision and Pattern Recognition (CVPR).

[39] Tan M., Le Q. EfficientNet: Rethinking model scaling for convolutional neural networks. Proceedings of the 36th International Conference on Machine Learning.

[40] d’Ascoli S., Touvron H., Leavitt M.L., Morcos A.S., Biroli G., Sagun L. Convit: Improving vision transformers with soft convolutional inductive biases. Proceedings of the 38th International Conference on Machine Learning.

[41] Deng J., Dong W., Socher R., Li L.-J., Li K., Fei-Fei L. ImageNet: A large-scale hierarchical image database. Proceedings of the 2009 IEEE Conference on Computer Vision and Pattern Recognition (CVPR).

[42] Redmon J., Divvala S., Girshick R., Farhadi A. You only look once: Unified, real-time object detection. Proceedings of the IEEE Conference on Computer Vision and Pattern Recognition (CVPR).

[43] Redmon J., Farhadi A. (2018). YOLOv3: An incremental improvement. arXiv.

[44] Ge Z., Liu S., Wang F., Li Z., Sun J. (2021). YOLOX: Exceeding YOLO series in 2021. arXiv.

[45] Lin T.-Y., Maire M., Belongie S., Hays J., Perona P., Ramanan D., Dollár P., Zitnick C.L., Fleet D., Pajdla T., Schiele B., Tuytelaars T. (2014). Microsoft COCO: Common objects in context. Computer Vision—ECCV 2014.

[46] Chen L.-C., Papandreou G., Schroff F., Adam H. (2017). Rethinking atrous convolution for semantic image segmentation. arXiv.

[47] Piansaddhayanaon C., Santisukwongchote S., Shuangshoti S., Tao Q., Sriswasdi S., Chuangsuwanich E. (2023). ReCasNet: Improving consistency within the two-stage mitosis detection framework. Artif. Intell. Med..

